# Heat-Killed Lactic Acid Bacteria Inhibit Nitric Oxide Production via Inducible Nitric Oxide Synthase and Cyclooxygenase-2 in RAW 264.7 Cells

**DOI:** 10.1007/s12602-021-09781-9

**Published:** 2021-04-05

**Authors:** Chang-Ho Kang, Jin-Seong Kim, Hyemin Kim, Hye Min Park, Nam-Soo Paek

**Affiliations:** MEDIOGEN, Co., Ltd., Jecheon, 27159 Korea

**Keywords:** Heat**-**killed, Probiotics, Nitric oxide, Inducible nitric oxide synthase, Cyclooxygenase-2

## Abstract

Heat-killed lactic acid bacteria perform immunomodulatory functions and are advantageous as probiotics, considering their long product shelf-life, easy storage, and convenient transportation. In this study, we aimed to develop appropriate heat treatments for industrial preparation of probiotics with antioxidant activity. Among 75 heat**-**killed strains, *Lactococcus lactis* MG5125 revealed the highest nitric oxide inhibition (86.2%), followed by *Lactobacillus acidophilus* MG4559 (86.0%), *Lactobacillus plantarum* MG5270 (85.7%), *Lactobacillus fermentum* MG4510 (85.3%), *L. plantarum* MG5239 (83.9%), *L. plantarum* MG5289 (83.2%), and *L. plantarum* MG5203 (81.8%). Moreover, the heat**-**killed selected strains markedly inhibited lipopolysaccharide-induced nitric oxide synthase and cyclooxygenase-2 expression. The use of heat-killed bacteria with intact bio-functionality can elongate the shelf-life and simplify the food processing steps of probiotic foods, given their high stability. The antioxidant and immune-modulatory activities of the heat-killed strains selected in this study indicate a strong potential for their utilization probiotic products manufacturing.

## Introduction

Probiotics are defined as “living microorganisms that provide health benefits beyond inherent basic nutrition” when consumed in appropriate quantities [1, 2]. The beneficial effects of probiotics include prevention and treatment of diarrhea, systemic infections, inflammatory bowel disease, immunodeficiency, allergies, cancers, and cholesterolemia [3–5]. Functional food products containing probiotics have several therapeutic benefits including anticancer, hypoglycemic, antioxidant, and immunomodulatory effects [6, 7]. Therefore, the identification and isolation of new probiotic strains with health-promoting benefits have garnered immense interest in the medical and industrial sectors [7].

Inflammation is a complex response of vascular tissues to harmful stimuli such as pathogens, damaged cells, and stimulants. It is mediated by various signaling molecules produced by macrophages, monocytes, and mast cells. In chronically inflamed tissue, the stimulus is persistent; therefore, recruitment of monocytes is maintained, and existing macrophages are tethered in place. Macrophages are especially important in innate immunity, as they immediately respond to microbial infections. They can kill pathogens directly by phagocytosis and indirectly via secretion of pro-inflammatory cytokines such as tumor necrosis factor (TNF)-α, interleukin (IL)-1β, and IL-6 [8] as well as excess amounts of mediators such as nitric oxide (NO) and prostanoids in response to lipopolysaccharide (LPS). After stimulation with LPS, pro-inflammatory mediators, NO, and prostaglandin E_2_ (PGE_2_) are generated in abundance by inducible NO synthase (iNOS) and cyclooxygenase-2 (COX-2), respectively [9].

The radical produced from L-arginine through the action of NOS [10] is one of the most important inflammatory intermediates and plays a crucial role in biological processes, including neurotransmission, immune defense, and apoptosis. To date, three isoforms of NOS, based on their Ca^2+^ calmodulin dependence [11] or tissue type, have been identified. Among these isoforms, iNOS produces large amounts of NO when cells are stimulated with LPS and cytokines (TNF-α, IL-1β, IFN-γ), which is further associated with the generation of potent reactive radicals, such as peroxynitrite [12]. Chronic inflammation can contribute to inflammatory pathologies by killing even healthy host cells by NO [13]. COX has two isoforms, COX-1 and COX-2, which convert arachidonic acid to prostaglandins. Similar to iNOS, COX-2 is an inducible form that produces proinflammatory PGs in inflammatory site [14]. As the eicosanoids play a pivotal role in inflammation, pain, and fever, the modulation of iNOS and COX-2 overproduction might represent a therapeutic goal in numerous inflammatory pathologies.

Therefore, in the present study, we aimed to evaluate the antioxidant with inflammation potential of heat-killed lactic acid bacteria (LAB) isolated from human origin and fermented food. The effect of heat-killed selected strains on the expression of proinflammatory mediators and cellular signaling pathways was investigated in LPS-induced murine macrophage, RAW 264.7 cells.

## Materials and Methods

### Sample Preparation

In this study, 75 LAB were isolated from humans and fermented food [15]. Isolated strains were identified by the 16S rRNA gene sequencing method (SolGent Co., Ltd. Korea). The selected strains were cultivated and maintained in MRS broth (Difco Laboratories, USA) at 37 °C. To evaluate the inflammation potential of these strains, overnight cultivated selected strains were heat-killed at 90 °C for 30 min. Following centrifugation (12,000×*g*, 5 min), cell pellets were rinsed thrice with phosphate-buffered saline (PBS) and suspended in Dulbecco’s Modified Eagle’s Medium (DMEM, BD Biosciences, Frankin Lakes, NJ, USA) to obtain concentrations of 5 × 10^8^ cells/mL by adjusting the absorbance at 600 nm wavelength.

## Cell Culture

The murine macrophage RAW 264.7 cell line was obtained from the Korean Cell Line Bank (KCLB, Korea) and maintained in DMEM (Gibco, NY, USA) supplemented with 10% fetal bovine serum (FBS; Gibco, NY, USA) and 1% penicillin/streptomycin (Gibco, NY, USA) at 37 °C in an atmosphere of 5% CO_2_. Cells were sub-cultured and plated at 80–90% of confluency.

## NO Production and Cell Viability

RAW 264.7 macrophage cells were grown at 37 °C and 5% CO_2_ in fully humidified air and sub-cultured every 3 days to 95% confluency. For routine subcultures, DMEM was supplemented with 10% FBS, penicillin (100 units/mL), and streptomycin (100 μg/mL). NO formation was detected based on the accumulation of nitrite, an indicator of NO synthesis, in the culture medium via the Griess reaction [16]. RAW 264.7 cells were plated at 2 × 10^5^ cells/well in a 96-well plate and stimulated with 1 μg/mL LPS, followed by the addition of isolated bacterial strains (10^7^ cells/well). After 24 h of incubation, NO concentration was determined by measuring the amount of nitrite in the cell culture supernatant using the Griess reagent. An absorbance measurement at 550 nm wavelength was obtained using the Epoch 2 microplate reader (BioTek, USA). Fresh culture medium was used as the blank control for all experiments.

3-[4,5-Dimethylthiazole-2-yl]-2,5-diphenyltetrazolium bromide (MTT; Sigma, USA) assay was performed to determine the viability of RAW 264.7 cells treated with the strains. RAW 264.7 cells were washed twice with PBS and 100 μL of MTT reagent (0.5 mg/mL) dissolved with PBS was added to each well. After 1 h of incubation, the MTT reagent was discarded and 100 μL of dimethyl sulfoxide (DMSO; Sigma, USA) was added to dissolve the formazan formed as a reactant between the MTT reagent and metabolites of live cells. The absorbance (A) was measured at 570 nm wavelength, and cytotoxicity was calculated in comparison with the result of a negative control group as follows.

Cell viability (%) = (A sample / A negative control) × 100.

## In Vitro Antioxidant Properties of the Selected Strains

The 2,2-diphenyl-1-picrylhydrazyl (DPPH; Sigma, USA) radical scavenging assay was performed according to Blois [17], with slight modifications. Briefly, the selected strains adjusted to an OD_600_ of approximately 1.0 with PBS (pH 7.4) were added to 0.05 mM DPPH solution (1:2 *v*/*v*) and mixed well. Thereafter, the mixtures were kept at room temperature for 30 min in the dark. The control reaction was prepared using ethanol added to the DPPH solution. The absorbance of each mixture was quantified at 517 nm wavelength. Each sample assay was performed in triplicate. The results were compared with those of ascorbic acid (10 μg/mL), and the antioxidant activity was calculated using the following formula: Scavenging effect (%) = (Ac-As)/Ac × 100, where As is the absorbance of the test sample and Ac is the absorbance of the control at 517 nm wavelength.

Scavenging of the 1 2,2′-azino-bis(3-ethylbenzothiazoline-6-sulfonic acid) (ABTS; Sigma, USA) radical was measured according to the method reported by Re et al. [18]. Briefly, the radical cation was prepared by mixing 7 mM of ABTS with 2.45 mM potassium persulfate (1:1 *v*/*v*), and the mixture was kept at room temperature in the dark for 24 h. Thereafter, 50 μL of the selected strain samples and 100 μL of ABTS solution were mixed and incubated for 10 min at room temperature. The absorbance of the mixture was measured at 734 nm wavelength. Each sample assay was performed in triplicate, and the scavenging rate was calculated as follows: scavenging rate (%) = (Ac-As)/Ac × 100, where As is the absorbance of the test sample and Ac is the absorbance of the control at 734 nm.

## Semi-quantitative Reverse Transcriptase-Polymerase Chain Reaction

Semi-quantitative reverse transcriptase-polymerase chain reaction (RT-PCR) was performed to determine the expression of COX-2 and iNOS mRNA. Total RNA was extracted from RAW 264.7 cells using TRI REGENT™ (Sigma Chemical Co., St. Louis, MO), according to the manufacturer’s recommendation. iNOS and COX-2 primers were designed for RT-PCR. Glyceraldehydes-3-phosphate dehydrogenase (GAPDH; Sigma, USA) was used as a housekeeping gene to normalize all samples. Table 1 lists the sequences of primer pairs used to amplify iNOS, COX-2, and GAPDH. RT-PCR was performed using the ONE-STEP RT-PCR PreMix kit™ (Qiagen Inc. Valencia, CA, USA), according to the manufacturer’s instructions. Each of the primers and 1 μg of the RNA template were mixed with ONE-STEP RT-PCR PreMix™. These samples were processed by one-step RT-PCR, under the following conditions: predenaturation of RNA at 95 °C for 5 min; 40 cycles of 95 °C for 15 s, 61 °C for 30 s, and 61 °C for 30 s; and a final elongation step of 30 s at 61 °C. The extent of iNOS and COX-2 mRNA expression was quantified using a densitometer with Quantity One software (Bio-Rad Lab., Hercules, CA, USA).

**Table 1:** Sequences of the primers used for RT-PCR assays

Gene		Sequence (5′ → 3′)	Length	Tm	GC%
iNOS	F	ACCATGGAGCATCCCAAGTA	20	58.40	50
	R	CCATGTACCAACCATTGAAGG	21	56.85	47.62
COX-2	F	AGCATTCATTCCTCTACATAAGC	23	56.47	39.13
	R	GTAACAACACTCACATATTCATACAT	26	55.90	30.77
GAPDH	F	TTGTCTCCTGCGACTTCAACA	21	59.86	47.62
	R	GCTGTAGCCGTATTCATTGTCATA	24	59.01	41.67

## Statistical Analysis

Results are expressed as means ± S.D. of three experiments. Difference between groups was evaluated using the Student’s *t* test, and a *P* value of < 0.05 was considered as statistically significant.

## Results and Discussion

### NO Productive Capacity and Cell Viability of Heat-Killed LAB

NO is a multi-functional mediator and plays a pivotal role in the immune response to inflammatory activity. The physiological or normal NO production in phagocytes is beneficial for the host defense against microorganisms, parasites, and tumor cells [19]. According to the results of the NO assay, the probiotic strains revealed a wide range of NO production inhibition rates (Table 2). This result indicated that bacterial strains would have different functional properties, even if they belong to the same species. Among the 75 probiotic strains, *Lac. lactis* MG5125 (86.2%) exhibited highest NO inhibition in LPS-induced cells, followed by *L. acidophilus* MG4559 (86.0%), *L. plantarum* MG5270 (85.7%), *L. fermentum* MG4510 (85.3%), *L. plantarum* MG5239 (83.9%), *L. plantarum* MG5289 (83.2%), and *L. plantarum* MG5203 (81.8%) (Table 2).

**Table 2:** Inhibitory activity of heat-killed lactic acid bacteria on NO production by LPS-induced RAW 264.7 macrophages

Origin	Isolated strains	Inhibition rate (%)^a^
Breast milk	*L. gasseri* MG4503	62.2 ± 0.5
	*L. gasseri* MG4506	65.0 ± 0.6
	*L. gasseri* MG4508	48.9 ± 1.7
	*L. gasseri* MG4512	30.7 ± 2.9
Human	*L. plantarum* MG4215	3.2 ± 1.3
	*L. plantarum* MG4221	16.4 ± 0.2
	*L. gasseri* MG4243	30.3 ± 1.2
	*L. fermentum* MG4254	− 27.4 ± 1.93
	*L. fermentum* MG4258	16.7 ± 1.5
	*L. fermentum* MG4261	− 48.3 ± 2.5
	*L. rhamnosus* MG4289	− 2.8 ± 1.3
	*L. rhamnosus* MG4298	8.3 ± 0.8
	*L. paracasei* MG4272	38.4 ± 1.5
	*L. plantarum* MG4229	53.1 ± 0.4
	*L. plantarum* MG4296	58.7 ± 1.5
Infant feces	*L. fermentum* MG4510 (in this study)	85.3 ± 0.2
	*L. fermentum* MG4532	63.9 ± 1.2
	*L. fermentum* MG4534	41.6 ± 1.1
	*L. plantarum* MG4553	66.5 ± 0.9
	*L. plantarum* MG4555	69.4 ± 1.4
	*L. plantarum* MG4556	71.4 ± 0.3
	*L. fermentum* MG4530	− 293.8 ± 2.0
	*L. fermentum* MG4531	− 255.1 ± 1.0
	*L. fermentum* MG4535	3.1 ± 0.3
	*L. fermentum* MG4536	− 83.2 ± 6.0
	*L. fermentum* MG4538	− 256.8 ± 1.8
	*L. fermentum* MG4539	− 180.6 ± 2.3
	*L. fermentum* MG4540	− 263.0 ± 1.0
	*L. gasseri* MG4520	− 32.1 ± 1.3
	*L. gasseri* MG4521	− 93.7 ± 2.1
	*L. gasseri* MG4524	49.6 ± 1.5
	*L. acidophilus* MG4559 (in this study)	86.0 ± 0.1
	*L. gasseri* MG4513	65.0 ± 0.1
	*L. gasseri* MG4514	54.5 ± 1.3
	*L. fermentum* MG4542	40.2 ± 1.9
	*L. salivarius* MG4527	− 71.4 ± 2.4
	*L. fermentum* MG4543	61.6 ± 1.4
	*L. fermentum* MG4544	46.1 ± 1.9
	*L. fermentum* MG4545	− 127.2 ± 5.4
Fermented food	*L. bulgaricus* MG5166	− 4.6 ± 3.9
	*L. salivarius* MG5212	− 18.2 ± 5.5
	*L. plantarum* MG5254	65.1 ± 0.3
	*L. plantarum* MG5270 (in this study)	85.7 ± 0.2
	*L. plantarum* MG5287	63.7 ± 1.4
	*L. plantarum* MG5289 (in this study)	83.2 ± 0.6
	*L. plantarum* MG5324	72.1 ± 1.3
	*L. plantarum* MG5143	79.1 ± 0.2
	*L. plantarum* MG5155	62.0 ± 1.8
	*L. plantarum* MG5197	78.4 ± 0.7
	*L. plantarum* MG5203 (in this study)	81.8 ± 0.1
	*L. plantarum* MG5239 (in this study)	83.9 ± 0.5
	*L. fermentum* MG5341	− 252.8 ± 3.8
	*L. paracasei* MG5135	− 161.6 ± 3.3
	*L. paracasei* MG5178	− 229.0 ± 2.1
	*L. paracasei* MG5189	− 6.6 ± 1.5
	*L. paracasei* MG5219	− 252.8 ± 1.4
	*L. paracasei* MG5310	− 214.5 ± 3.1
	*L. casei* MG5275	− 146.7 ± 3.2
	*L. casei* MG5296	− 64.7 ± 0.5
	*Lac. lactis* MG5124	79.2 ± 0.1
	*Lac. lactis* MG5125 (in this study)	86.2 ± 0.3
	*Lac. lactis* MG5128	60.3 ± 1.0
	*Lac. lactis* MG5129	79.7 ± 0.1
	*Lac. lactis* MG5278	62.3 ± 0.1
	*S. thermophilus* MG5152	2.6 ± 6.2
	*S. thermophilus* MG5304	68.1 ± 2.4
	*S. thermophilus* MG5343	25.9 ± 0.7
	*S. thermophilus* MG5344	22.0 ± 5.3
	*S. thermophilus* MG5150	62.9 ± 0.6
	*L. helveticus* MG5161	66.2 ± 0.9
	*L. helveticus* MG5162	16.2 ± 1.3
	*L. helveticus* MG5220	16.3 ± 1.0
	*L. helveticus* MG5290	47.9 ± 0.5
	*L. delbrueckii* subsp. *bulgaricus* MG5165	− 52.8 ± 1.6
	*L. delbrueckii* subsp. *bulgaricus* MG5168	− 36.9 ± 4.6

*L.* *Lactobacillus*, *Lac.* *Lactococcus*, *S.* *Streptococcus*

^a^Average ± SD (*n* = 3)

The heat-killed selected strains showed low toxicity in RAW 264.7 cells, with cell viability of approximately 82.06–111.66%. The effect of killed selected strains on cell viability increased in a dose-dependent manner (Table 3).

**Table 3:** Viability of cells treated with heat-killed selected strains at different concentrations

Selected strains	Cell viability (%)	
	^a^2 × 10^7^	2 × 10^8^
*L. acidophilus* MG4559	94.65 ± 4.80	101.20 ± 3.64
*L. fermentum* MG4510	99.20 ± 3.50	111.66 ± 3.85
*L. plantarum* MG5203	84.17 ± 4.16	82.06 ± 4.79
*L. plantarum* MG5239	94.26 ± 3.54	76.89 ± 3.14
*L. plantarum* MG5270	106.41 ± 2.91	88.66 ± 4.05
*L. plantarum* MG5289	96.21 ± 3.33	83.85 ± 2.19
*Lac. lactis* MG5125	108.02 ± 3.16	103.34 ± 3.78

^a^Heat-killed selected strains with different cell concentrations (cells/mL)

## In Vitro Antioxidant Properties of the Selected Strains

**Fig. 1:**
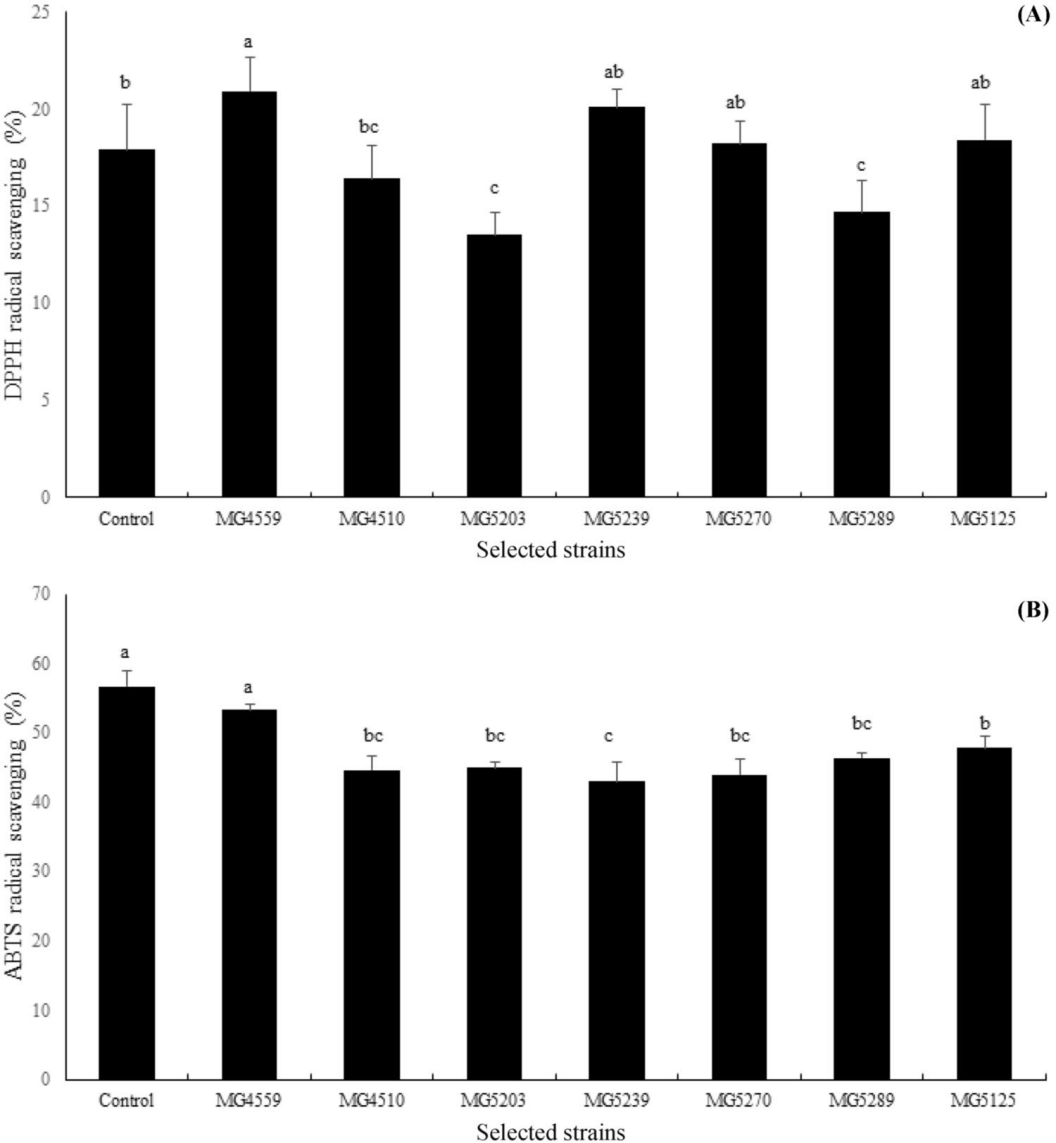
DPPH radical scavenging ability (**a**) and ABTS radical scavenging ability (**b**) of the selected strains in this study. Different letters (a–c) indicate significant difference at *p* < 0.05

The antioxidant properties of the four selected strains, which revealed high inhibition of NO production, were evaluated by DPPH and ABTS radical scavenging activities. The DPPH free radical scavenging activities of the probiotic strains ranged from 14.7 to 20.9% (Fig. 1a). *L. acidophilus* MG4559 exhibited the highest radical scavenging activity (20.9%) and similar antioxidant activity when compared with that of ascorbic acid (10 μg/mL) control (17.9%), followed by *L. plantarum* MG5239 (20.1%). Regarding ABTS radical scavenging activities, results of the strains ranged from 43.0 to 53.3% (Fig. 1b). *L. acidophilus* MG4559 exhibited the highest radical scavenging activity (53.3%) and similar antioxidant activity when compared with that of ascorbic acid (10 μg/mL) control (56.7%), followed by *Lac. lactis* MG5125 (47.8%).

All seven strains presented high antioxidant activities, indicating that the selected probiotic strains possess the ability to reduce ROS. The trends of antioxidant activity and NO production inhibition rate were similar. Our results were in accordance with those of other studies regarding antioxidant activities of *Lactobacilli*. Li et al. [21] reported the antioxidant activities of *L. plantarum* strains derived from food, and Afify et al. [22] reported the ABTS radical scavenging effects of *L. reuteri*. Lin and Yen [23] evaluated the inhibitory effect of *Bifidobacterium longum*, and Kim et al. [24] isolated antioxidative *Bifidobacterium* species from infant fecal samples. Notably, probiotics produce bioactive compounds with beneficial properties, including antioxidant activity, and may act via specific molecular mechanisms responsible for defense against oxidative stress based on the strain specificity [20, 25].

## Immunomodulatory Activity of Heat-Killed Selected Strains on Murine Macrophage RAW 264.7 Cells Via RT-PCR

**Fig. 2:**
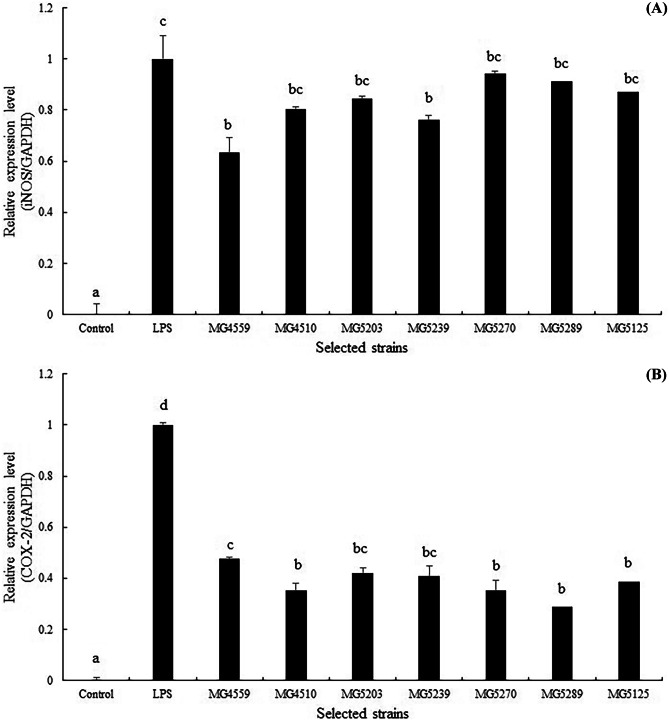
RT-PCR analysis of mRNA expression of iNOS (**a**) and COX-2 (**b**). Glyceraldehyde-3-phosphate dehydrogenase (GAPDH) was used as a housekeeping gene to normalize all samples. Data are representative of three experiments, respectively. Values represented indicate the means ± SD of three independent experiments. Different letters (a–d) indicate significant difference at *p* < 0.05

To evaluate the immunomodulatory ability, RT-PCR assays were performed. Cells were treated with heat-killed bacteria as described earlier, and the results are illustrated in Fig. 2. The iNOS and COX-2 gene expression markedly increased following LPS stimulation; however, heat-killed selected strains remarkably inhibited LPS-induced iNOS (Fig. 2a) and COX-2 (Fig. 2b) expression. The expression of the housekeeping gene GAPDH was not affected by the heat-killed selected strains. Raw 264.7 macrophages are representative antigen-presenting cells (APCs); they participate in the first stage of innate immunity by swallowing pathogens and inducing the release of intercellular signaling molecules, such as NO and COX-2. When the body’s immune system confronts immune-challenging elements, these cells rapidly initiate colonization, secrete cytokines, and activate natural killer cells and dendritic cells [26]. NO, induced by iNOS and cytokines, acts as a protective agent against pathogens and decreases their leukocyte adherent-activity [27]. In previous studies, the immuno-modulatory abilities of polysaccharides in cell walls have been reported by several researchers. Immunomodulatory polysaccharides activate innate and adaptive immune responses via direct and indirect interactions. Since probiotics are equipped with varying compositions of intrinsic enzymes, their immunomodulatory properties could be strain-specific [28].

## Conclusion

We aimed to select superior probiotic strains with desired antioxidant activity from 75 strains of probiotic candidates obtained from human origin and fermented foods by evaluating their inhibitory activity on NO production. In this study, molecular mechanisms were not elucidated and the heat-killed strains metabolites related to the inhibition of NO production should be investigated. However, we selected seven probiotic strains that exhibited high antioxidant activities. The use of heat-killed cells, which still maintain their bio-functionality, can elongate the shelf-life and simplify the food-processing steps of probiotic foods, given their high stability. Recently, LAB have been studied to prevent and treat inflammatory conditions in vivo and in vitro. These positive effects might be related to direct and indirect molecular mechanisms. Finally, the antioxidant and immunomodulatory activities of the heat-killed strains selected in this study indicate strong potential for their utilization in probiotic product manufacturing.

## Data Availability

The authors declare that all data and materials support published claims and comply with field standards.
